# Training data size and predication errors in the use of machine-learning assisted intraocular lens power calculation

**DOI:** 10.1038/s41598-023-38616-6

**Published:** 2023-07-13

**Authors:** Hitoshi Tabuchi, Tomofusa Yamauchi, Tomohiro Shojo, Kosuke Takase, Mao Tanabe

**Affiliations:** 1 Department of Technology and Design Thinking for Medicine, Hiroshima University, 1-2-3 Kasumi, Minami-ku, Hiroshima, 734-8553 Japan; 2 Department of Ophthalmology, Tsukazaki Hospital, 68-1 Waku, Aboshi-ku, Himeji, Hyogo 671-1227 Japan

**Keywords:** Eye diseases, Lens diseases, Biomedical engineering

## Abstract

This retrospective study examined the effect of the size of training data on the accuracy of machine learning-assisted SRK/T power calculation. Clinical records of 4800 eyes of 4800 Japanese patients with intraocular lenses (IOLs) were reviewed. A support vector regressor (SVR) was used for refining the SRK/T formula, and dataset sizes for training and evaluation were reduced from full to 1/64. The prediction errors from the postoperative refractions were calculated, and the proportion within ± 0.25 D, ± 0.50 D, and ± 1.00 D of errors were compared with those using full data. The influence of the difference in A-constant was also evaluated. Prediction errors within ± 0.50 D in the use of full data were obtained with the dataset of ≥ 150 eyes (*P* = 0.016), whereas the datasets of ≥ 300 eyes were required for the error within ± 0.25 D (*P* < 0.030). The prediction errors did not alter with the A-constant values among IOLs with open-loop haptics, except for IOLs with plated haptics. In conclusion, the accuracy of SVR-assisted SRK/T could be achieved with the training dataset of ≥ 150 eyes for the Japanese population, and the calculation was versatile for any open-looped IOLs.

## Introduction

In the use of premium intraocular lenses (IOLs) for astigmatism and presbyopia corrections, accurate IOL power calculation for postoperative emmetropia is necessary for IOL functions. Although postoperative refractive errors within ± 1.0 D could be obtained in 93% of eyes using third- to fourth-generation calculation formulas such as the SRK/T and Haigis formula^1^, accuracy of > 90% within the absolute errors of 0.5 D is desired for patients undergoing premium IOL implantation. Thus, advanced calculation methods, such as the Barrett Universal II (BUII)^2^, Hill-radial basis function (Hill-RBF)^3^, and Kane formula^4^, have been used, and several publications have reported their superiority^5–7^. New-generation formulas enable higher accuracy by adding more biometric measurements such as lens thickness and corneal diameter, utilizing a complex model of ocular geometry, and utilizing machine learning with a large dataset.

As most of the advanced calculations are based on the biometry of Caucasian eyes, performances could be inherently altered by patients’ ethnicity, race, and region. The alternations for patient groups of a site have been adjusted with the constants of third- to fourth-generation formulas, such as the A-constant. However, such optimization is not available for advanced calculations^8^. Recently, we demonstrated that the use of machine learning with the SRK/T formula effectively improved the power calculation accuracy for a patient group^9^. Predicted refractions derived from the SRK/T formula were adjusted with support vector regression (SVR) machine learning. The SVR nonlinearly provided a regression equation in which the total errors of the training data outside a certain margin from the equation were minimized^10^ and were suitable for IOL power calculation^11^. With training data of 1211 eyes, the prediction errors were less than that with BUII for patients in the Kyushu Island of Japan^9^. Adaptation was achieved using a small size of training data by incorporating SRK/T; however, how much training data are required for a specific accuracy is not certain. Thus, this retrospective study aimed to assess the effect of training data size on the accuracy of IOL power calculation and evaluate the influence of the difference in A-constants.

## Methods

### Participates

This retrospective study was approved by the Institutional Ethics Committee of Tsukazaki Hospital (Approval No. 181011) and adhered to the tenets of the Declaration of Helsinki. For all participants, the use of clinical records related to cataract surgery was approved as stated in the informed consent obtained before surgery. Clinical records of consecutive patients who underwent cataract surgery with IOL implantation between September 2017 and April 2021 were reviewed. The inclusion criteria were as follows: no history of refractive surgery, postoperative corrected distance visual acuity (CDVA) of 16/20 in Snellen or better, and optimized constants of implanted IOLs. For bilateral implantation, an eye with regular and mild astigmatism was selected for the analysis.

Preoperative axial length (AXL), corneal radius (CR), anterior chamber depth (ACD), lens thickness (LT), and white-to-white distance (WTW) were measured using a swept-source biometer IOLMaster 700 (Carl Zeiss, Oberkochen, Germany). IOL power was determined using the SRK/T formula, and all IOLs were implanted in the capsule without complications. Three months after surgery, the manifest refraction spherical equivalent (MRSE) was measured during the examination for CDVA.

### Machine learning-assisted power calculation

SVR was used to enhance the accuracy of the SRK/T formula^9^. Initially, predicted postoperative refractions were obtained using the SRK/T with biometry measurements of AXL and CR and an optimized A-constant. The predicted postoperative refractions were refined for the patient group with additional inputs of AXL, CR, ACD, LT, and WTW. The SVR with an RBF kernel was trained by using the “scikit-learn” library (https://scikit-learn.org/stable/modules/svm.html#svm-regression) in Python 3. Hyperparameters such as the C-constant and shape parameter γ of the kernel function were tuned using a grid search for avoiding overfitting^11^.

**Figure 1:**
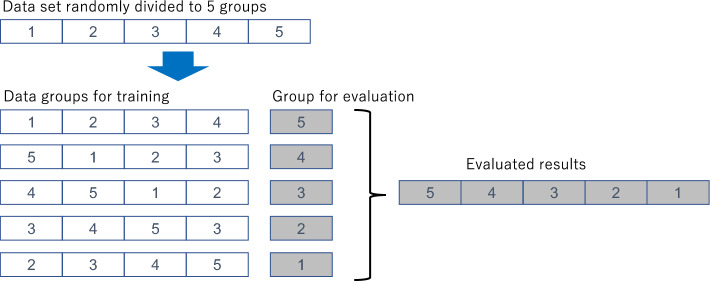
Cross-validation of the effect of dataset size. The dataset was randomly divided into five groups. Combinations of four groups were assigned for training the SVR-assisted SRK/T power calculation, and the prediction errors were obtained from the rest of them.

To evaluate the effect of training data sizes on calculation accuracy, a dataset of the participants was randomly divided into five groups. Initially, four groups were used for SVR training to refine the accuracy of the predicted postoperative refractions, and the remaining group was used to evaluate the trained calculator. As shown in Fig. 1, the groups used for training were rearranged four times to obtain evaluation results for all data. Then, size of the dataset was reduced by half and divided into five groups, and training and evaluation were conducted similarly. The dataset had been divided by two until the size was 1/64 of the original size. When the original data size was 4800 eyes, training and evaluation were conducted with datasets of 4800, 2400, 1200, 600, 300, 150, and 75 eyes.

### Analysis

To assess the accuracy for each training data size from the SVR, prediction errors of the predicted postoperative refractions from MRSE were obtained, and its means and standard deviations (SDs) were calculated. The median of the absolute prediction error (MedAE) was also obtained. Changes in the mean prediction errors with the dataset sizes were examined using the analysis of variance (ANOVA), followed by Holm’s multiple comparisons in the presence of a significant change. The proportion of eyes within prediction errors ± 0.25 D, ± 0.50 D, and ± 1.00 D was calculated, and differences from the use of full data were examined using the Chi-squared test.

The influence of eyes with long AXL (> 26.0 mm) was also compared with those of eyes with normal AXL (between 22.0 and 26.0 mm). Owing to the limited sample size (178 eyes), eyes with short AXL (< 22.0 mm) were not analyzed. The prediction errors were compared using a t-test, and proportions within ± 0.25 D, ± 0.50 D, and ± 1.00 D errors were compared using the Chi-squared test.

To investigate whether the calculator would accommodate various IOL models, the influence of A-constants on prediction errors was also evaluated. Prediction errors were compared between four groups according to the ranges of A-constants, such as ≤ 119.0, 119.0–119.2, 119.2–119.4, and 119.4–119.6, using ANOVA following the Tukey multiple comparison. *P* < 0.05 was considered a statistically significant difference.

### Ethics approval and consent to participate

This retrospective study was approved by the Institutional Ethics Committee of Tsukazaki Hospital (Approval No. 181011) and adhered to the tenets of the Declaration of Helsinki. For all participants, the use of clinical records related to cataract surgery was approved as stated in the informed consent obtained before surgery.

### Consent to publish

Name and other personally identifiable information were removed from all text/figures/tables/images.

## Results

Clinical records of 4800 eyes from 4800 eligible patients were available. The mean age of the patients was 71.5 (SD 8.4) years, and there were 2195 men and 2605 women. The preoperative mean AXL, CR, and ACD were 24.0 (SD 1.5; range 20.5–30.5) mm, 7.63 (SD 0.26; range 6.72–8.54) mm, and 3.11 (SD 0.40; range 1.75–4.62) mm, respectively. The LT and WTW were 4.53 (SD 0.46) mm and 11.7 (SD 0.4) mm, respectively. The implanted IOLs and A-constants used are listed in Table 1. The power of the implanted IOLs ranged from 5.0 to 30.0 D, and the mean power was 19.4 (SD 4.0) D for targeting refractions between − 7.42 D and 1.13 D (mean − 0.20 D). The mean postoperative MRSE was − 0.18 (SD 0.90) D, and the CDVA was − 0.11 (SD 0.08) logMAR.

**Table 1:** Demographic data of subjects.

Intraocular lens model	Type	No of eyes	A-constant used
CP2.2, YP2.2 (Kowa)	Mono	958	119.12
DFR00V & DFW150/225/300/375 (J&J)	EDF	29	119.07
KS-SP (Staar)	Mono	32	119.55
LS-313 MF15, LS-313 MF15T (Oculentis)	EDF	603	118.26
NX-70 (Santen)	Mono	1	119.33
SN60WF, SN6AT3-9 (Alcon)	Mono	359	119.21
SN6AD1 (Alcon)	Bi	26	119.16
SND1T3-6 (Alcon)	Bi	50	119.21
SV25T3-6 (Alcon)	EDF	50	119.48
SY60WF (Alcon)	Mono	28	119.16
SZ-1/C (Nidek)	Mono	289	119.45
TFNT00/30/40/50/60 (Alcon)	Tri	299	119.31
W-60R (Santen)	Mono	164	119.51
X-70 (Santen)	Mono	30	119.33
XC1, XY1, XY1AT3-7 (Hoya)	Mono	68	119.18
YP2.2 (Kowa)	Mono	886	119.12
ZCB00, ZCB00V (J&J)	Mono	505	119.39
ZCT150/225/300/400, ZCV150/225/300/375, ZCW150/225/300/375 (J&J)	Mono	645	119.46
ZKB00, ZLB00, ZMB00 (J&J)	Bi	466	119.60
ZXR00V, ZXV150/225/300/375, ZXW300 (J&J)	EDF	198	119.23

*Mono* monofocal IOL with and without toric, *EDF* extended depth-of-focus IOL with and without toric, 3: *Bi* bifocal IOL with and without toric, *Tri* trifocal IOL with and without toric.

**Figure 2:**
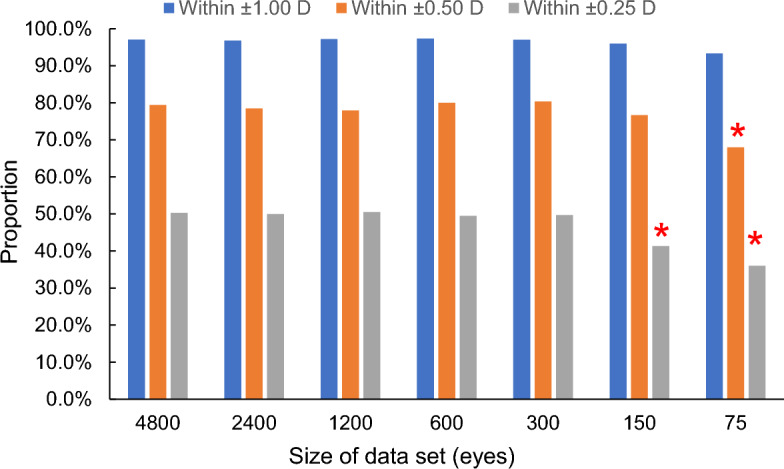
Effect of the dataset sized from full (4800 eyes) to 75 eyes on the proportions within ± 0.25 D, ± 0.50 D, and ± 1.00 D errors. Compared with the use of overall data (N = 4800), the proportions within ± 0.50 D error for the dataset of 75 eyes were significantly less (*P* = 0.016, * in Fig.). For errors within ± 0.25 D, the use of datasets of 75 and 150 eyes resulted in a lower proportion (*P* < 0.030, * in Fig.).

Table 2 shows the mean prediction errors, MedAE, and proportions within ± 0.25 D, ± 0.50 D, and ± 1.00 D errors in the use of SVR-assisted calculation for seven dataset sizes. The mean prediction errors did not change with the data size (*P* = 1.00, ANOVA), whereas the SD values increase compared with the overall data of 4800 when the data size were ≤ 300 (*P* < 0.027, F-test). Figure 2 shows the change in proportions within ± 0.25 D, ± 0.50 D, and ± 1.00 D errors with the dataset size. Compared with the results using overall data, the proportions within ± 0.50 D error for the dataset of 75 eyes were significantly low (*P* = 0.016, Chi-squared test). For errors within ± 0.25 D, the use of datasets of 75 and 150 eyes resulted in a lower proportion (*P* = 0.014 and 0.030, respectively). In comparison with the results of SRK/T only (N = 4800 eyes), the proportion within ± 0.50 D error was higher when the dataset size was ≥ 150, whereas it was lower for the size of 75 (*P* < 0.001).

**Table 2:** Refractive errors of SVR-assisted calculations with data set sizes from 4800 to 75 eyes.

Date set/training data size (eye)	4800/3840	2400/1920	1200/960	600 80	300/240	150/120	75/60
Mean (SD), D	0.00 (0.44)	− 0.01 (0.46)	− 0.01 (0.47)	− 0.01 (0.46)	0.00 (0.43)	0.00 (0.52)	0.01 (0.59)
MedAE, D	0.25	0.25	0.25	0.25	0.26	0.31	0.31
Within ± 0.25 D	50.3%	50.0%	50.5%	49.5%	49.7%	41.3%	36.0%
Within ± 0.50 D	79.4%	78.5%	77.9%	80.0%	80.3%	76.7%	68.0%
Within ± 1.00 D	97.0%	96.8%	97.2%	97.3%	97.0%	96.0%	93.3%

*D* diopter, *MedAE* medium of absolute prediction errors, *SD* standard deviation.

The influence of long eyes (AXL > 26.0 mm) was evaluated in comparison with normal eyes (AXL of 22.0–26.0 mm). Table 3 lists refractive errors for long and normal eyes. In the mean prediction errors, no differences were found for all dataset sizes (*P* > 0.19, t-test). Within ± 0.25 D, ± 0.50 D, and ± 1.00 D errors, the proportions in long eyes were significantly less for a dataset size of 75 eyes (*P* < 0.003).

**Table 3:** Refractive errors of SVR-assisted calculations for long and normal eyes.

Date set size (eye)	4800	2400	1200	600	300	150	75
Mean (SD), D							
Long AXL	− 0.03 (0.48)	− 0.02 (0.49)	− 0.03 (0.49)	− 0.03 (0.50)	− 0.03 (0.51)	− 0.02 (0.54)	− 0.01 (0.71)
Normal AXL	0.00 (0.43)	0.00 (0.43)	0.00 (0.43)	0.00 (0.44)	0.00 (0.44)	0.00 (0.46)	0.01 (0.49)
Within ± 0.25 D error							
Long AXL	50.9%	48.5%	50.9%	48.3%	46.8%	45.0%	39.6%
Normal AXL	50.4%	50.2%	50.5%	49.9%	49.1%	48.0%	47.0%
Within ± 0.50 D error							
Long AXL	78.9%	78.2%	77.6%	78.2%	75.8%	74.9%	69.2%
Normal AXL	79.7%	79.0%	79.0%	79.4%	78.1%	77.5%	75.2%
Within ± 1.00 D error							
Long AXL	96.9%	97.1%	97.3%	96.7%	96.5%	95.1%	92.2%
Normal AXL	97.2%	97.1e%	97.2%	97.1%	96.9%	96.3%	95.3%

D: diopter, SD: standard deviation, AXL: axial length.

Changes in the prediction error with the A-constant used were examined. In this study, 603, 1109, 1442, and 1646 eyes had A-constants of implanted IOLs of ≤ 119.0, 119.0–119.2, 119.2–119.4, and 119.4–119.6, respectively. As seen in Table 1, the A-constants of ≤ 119.0 consisted of only a single type of IOLs (LS-313 MF15 and LS-313 MF15T) of hydrophilic acrylic material with plated haptics, whereas other IOLs had open-loop haptics with various materials. Table 4 shows the mean prediction errors, MedAE, and proportions within ± 0.25 D, ± 0.50 D, and ± 1.00 D errors. The mean prediction error for A-constants of ≤ 119.0 significantly shifted to hyperopia compared with A-constants of 119.2–119.4 (*P* = 0.0041, Tukey multiple comparisons), whereas no change was observed among IOLs of A-constants of 119.0–119.6.

**Table 4:** Refractive errors in the ranges of A-constants with the SVR-assisted calculations (Data set size of 4800 eyes).

A-constants	119.0 and less (N = 603)	119.0–119.2 (N = 1109)	119.2–119.4 (N = 1442)	119.4–119.6 (N = 1646)
Mean (SD), D	0.05 (0.55)	− 0.01 (0.41)	− 0.02 (0.43)	0.00 (0.42)
MedAE, D	0.03	0.00	− 0.01	0.00
Within ± 0.25 D	42.1%	52.3%	50.7%	51.6%
Within ± 0.50 D	69.5%	82.0%	80.1%	80.7%
Within ± 1.00 D	93.5%	97.5%	97.5%	97.6%

*D* diopter, *MedAE* medium of absolute prediction errors, *SD* standard deviation.

## Discussion

The use of SVR with the SRK/T formula improved the accuracy of IOL power calculations, and the accuracy did not degrade when the dataset size for SVR training was ≥ 150 within ± 0.50 D errors. The calculator was versatile for any IOLs with an open loop. In the analysis by Aristodemou et al. using data from 8180 eyes and conventional statistical techniques, data from 243 eyes would be required to optimize each A-constant, and the accuracy increases with the sample size^12^. In the current results, the accuracy remained until the dataset size of 300. This superior performance with a small sample size would result from the use of nonlinear SVR. In addition, the refining of SRK/T outputs accommodated the IOL with A-constants of 119.0–119.6. Previous assessments of machine-learning power calculations used multiple types of IOLs for trainings^11,13^; however, the difference in IOLs were not examined. Our results indicated that the calculator accommodated most of the one-piece hydrophobic acrylic IOLs with open haptics.

While the mean prediction errors and MedAE did not change with the dataset size, the variance increases when the size was ≤ 150. As a result, the accuracy within ± 0.25 D errors was lower when the dataset size was ≤ 150. For attaining high accuracy, data of ≥ 300 eyes would be preferred. Thanks to the accommodation of multiple IOL types for training, such dataset size would be acceptable for optimization for a patient group at each site or surgeon.

In the comparison between long and normal eyes, significant differences were found in the use of 75-eye dataset. Similarly, the use of small datasets results in lower performance in the proportion within ± 0.5 D and ± 0.25 D errors. One of the factors would be limited coverage of datasets; thus, accommodating eyes with long or short AXL and minor IOL design would be difficult. In the current analysis, ≥ 150 eyes were the least recommended for Japanese patients in the territory of the site. To provide favorable postoperative outcomes, collecting data from patients within each territory would be better.

In the comparison of A-constants, only a particular IOL type showed lower outcomes. This IOL was extended depth-of-focus, made of hydrophilic acrylic material, and equipped with plated haptics. Compared with other IOL types of one-piece and open-loop haptics, the mean prediction errors were significantly and slightly shifted to hyperopic. As the shifting of the IOLs posteriorly resulted in hyperopic errors^14^, bending of plated haptics due to capsule contraction would induce this prediction error. Further investigation is required. Except for a particular IOL model, the current machine learning-assisted power calculation improved the accuracy for the A-constants in the range of 119.0–119.6, whereas the training dataset insisted on data with multiple IOL models. This finding would be attributed to the use of SRK/T outputs and optimized A-constants; thus, optimized IOL power calculation for our patient group with a limited training dataset would be beneficial. Further investigation is necessary to verify this speculation.

This study has some limitations. First, owing to the retrospective design, the topographic data of the cornea was not measured. Refractive powers of the cornea were obtained from the powers of the anterior (keratometric) and posterior surfaces and corneal thickness. Thus, the influence of the posterior cornea could not be evaluated. Further evaluation with the use of a rotational Scheimpflug camera or optical coherence tomography^15^ is necessary for more accurate power calculation. In addition, the influence of the asphericity of the cornea^16^ should be examined. Moreover, multiple IOLs are available for training and evaluation. As per the guideline presented^8^, an IOL power calculation was evaluated for a single IOL model. In the previous assessment of the same calculation with 1611 eyes with SN60WF alone, the mean prediction error was 0.01 (SD, 0.38) D, and the proportions within ± 0.25 D, ± 0.50 D, and ± 1.00 D errors were 54.4%, 83.5%, and 98.5%, respectively^9^, which were slightly better than the current results. As expected, a higher accuracy would be obtained by selecting the IOL type routinely used. In other cases, the range of the dataset was determined by the biometry of limited patients within the territory. Hence, there would be patients who would be out of the range of the dataset used for the training. Ideally, a dataset includes a heterogeneous cohort of patients as much as possible; however, this is not practical. However, indicating the minimum requirement for a clinical situation would be important. Finally, implementing the proposed calculation in clinical practice is not easy, since the calculator works in Python 3. To examine the effectiveness of the proposed calculator in other sites, an environment in which a user-friendly calculator can be used through the web is warranted.

## Conclusions

This study using data from 4800 eyes revealed that the accuracy of SVR-assisted SRK/T power calculation could be achieved with the training dataset of ≥ 150 within ± 0.50 D errors for the Japanese population. The calculation was versatile for any open-looped IOL models.

## Data Availability

The datasets used and/or analyzed during the current study are available from the corresponding author upon reasonable request.
